# Mechanisms, Imaging Phenotypes, and Therapeutic Advances of Neovascularization in Brain Metastases

**DOI:** 10.3390/biomedicines14010119

**Published:** 2026-01-07

**Authors:** Siheng Liu, Bingyang Shan, Yiming Zhang, Lixin Xu, Xiaolei Zhang, Liguo Ye, Huantong Diao, Ye Cheng, Jie Tang

**Affiliations:** 1 Department of Neurosurgery, Xuanwu Hospital, Capital Medical University, Beijing 100053, China; 2 Department of Neurosurgery, China International Neuroscience Institute, Beijing 100053, China; 3 China Clinical Alliance of Intracranial Metastasis (CLAIM), Beijing 100053, China

**Keywords:** brain metastasis, neovascularization, angiogenesis, vessel co-option, vasculogenic mimicry, radiomics

## Abstract

Brain metastases have a distinctive vascular ecosystem—shaped by sprouting angiogenesis, vessel co-option, vasculogenic mimicry, and tumor cell transdifferentiation—that governs tumor perfusion, drug exposure, and therapeutic responsiveness. These heterogeneous vascularization patterns exhibit characteristic differences in enhancement morphology, perfusion levels, and metabolic uptake on contrast-enhanced MRI, perfusion imaging, and amino acid PET, providing crucial imaging cues for identifying routes of blood supply, inferring the state of the blood–tumor barrier, and guiding individualized therapeutic strategies. Anti-VEGF therapy is primarily used to alleviate cerebral edema and radiation necrosis, yet it confers limited survival benefit, underscoring the spatiotemporal heterogeneity of the blood–tumor barrier and the persistence of non-classical vascularization pathways. Building on the concept of “vascular normalization,” combinations of anti-angiogenic therapy with immunotherapy, radiotherapy, or targeted agents have shown encouraging intracranial activity in selected settings—most robustly in melanoma brain metastases—but remain insufficiently validated in randomized, brain-metastasis-focused trials. By integrating mechanistic, imaging, and therapeutic perspectives, this review outlines how vascular-ecosystem-based stratification and physics-informed drug-delivery strategies may help transition anti-vascular therapy from symptomatic control toward mechanism-driven precision intervention.

## 1. Introduction

Brain metastases (BM) represent the most common intracranial tumors in adults, occurring in approximately 20–40% of all cancer patients—far exceeding the total incidence of primary brain tumors [1]. The most frequent primary sources include lung cancer (≈40–50%), breast cancer (≈15–25%), melanoma (≈5–20%), and, to a lesser extent, colorectal and renal carcinomas, highlighting pronounced differences in the brain-tropism of various cancers [2,3]. Biologically, metastatic lesions within the brain are not mere replicas of their primary tumors. Instead, they undergo genetic and phenotypic reprogramming under clonal selection and the selective pressures of the brain microenvironment, giving rise to distinct evolutionary trajectories and immune ecologies. These “brain-specific subclones” often harbor independent resistance mechanisms and adaptive strategies [4,5].

Clinically, a well-recognized phenomenon is the discordance between intracranial and extracranial disease control. Even when intracranial lesions are adequately managed by local therapy, patients may succumb to extracranial progression; conversely, highly effective systemic therapy may control the primary tumor and extracranial metastases while failing to prevent new or subclinical intracranial lesions, which reside in a relative “pharmacologic sanctuary” [6,7]. As a result, CNS progression-free survival (CNS-PFS) often decouples from overall survival (OS). This has motivated recent clinical trials to adopt CNS-PFS and intracranial objective response rate (CNS-ORR) as primary or key secondary endpoints rather than relying solely on systemic PFS or OS, which may obscure the true lack of CNS benefit [8].

In the healthy brain, the blood–brain barrier (BBB) provides rigorous protection through tightly apposed endothelial cells, pericyte coverage, and astrocytes, collectively restricting the entry of most drugs and immune cells [8,9]. Once cancer cells colonize the brain, they migrate and spread along pre-existing microvessels—an event known as vessel co-option—progressively disrupting local barrier structures and contributing to the blood–tumor barrier (BTB). Although the BTB is more permeable than the BBB, its permeability is highly patchy and heterogeneous: some regions permit the entry of small molecules and even selected large molecules or immune cells, while others remain relatively sealed, creating pharmacologic and immunologic “blind spots.” This spatially heterogeneous BTB results in substantial intratumoral variation in drug accessibility [10,11,12].

Classical sprouting tumor angiogenesis—hypoxia-driven VEGF upregulation that stimulates sprouting of new capillaries—has long been considered a major contributor to tumor growth and symptomatic vasogenic edema in brain metastases. Consequently, anti-VEGF agents such as bevacizumab are widely used to alleviate radiation necrosis–related edema, reduce corticosteroid dependence, and improve neurological function [13,14]. However, in unstratified patient populations, VEGF inhibition alone rarely translates into durable OS benefit and fails to reliably prevent intracranial recurrence. Retrospective and small prospective studies to date have not demonstrated a major, consistent increase in intracranial hemorrhage with bevacizumab, although sample sizes are limited and individualized risk assessment remains essential, reinforcing its primary role as a symptomatic therapy rather than a curative strategy. The consistent gap between symptomatic improvement and sustained tumor control indicates that brain metastases do not rely solely on VEGF-driven sprouting angiogenesis. Instead, metastatic cells can maintain blood supply and evade immune elimination through alternative vascularization programs, including vessel co-option, vasculogenic mimicry, and tumor cell transdifferentiation into endothelial- or pericyte-like phenotypes [12,15,16,17]. These mechanisms—originally described in aggressive melanoma and glioblastoma and now increasingly recognized in brain metastases—will be discussed in detail in subsequent sections.

Against this backdrop of clinical and biological complexity, this review addresses three central questions: (1) What vascular supply patterns exist in brain metastases, and how do these patterns dynamically shift within the brain microenvironment? (2) What benefits can current vascular-targeted treatment strategies realistically achieve within the CNS compartment? (3) How do tumors remodel their vascular ecosystem under therapeutic pressure to develop resistance, and how can these insights guide a transition toward vascular ecosystem–based personalized management? In contrast to prior reviews that have focused primarily on either clinical management or basic vascular biology, we specifically integrate mechanistic, imaging, and therapeutic data into a unified “vascular ecosystem” framework for brain metastases.

## 2. Classical Sprouting Angiogenesis in Brain Metastases

### 2.1. The Hypoxia–HIF Axis Initiates Angiogenic Programs in Brain Metastases

Following rapid proliferation within the brain parenchyma, metastatic tumor cells often outstrip the local oxygen supply, giving rise to a profoundly hypoxic microenvironment. Tumor cells respond by stabilizing and accumulating the transcription factors HIF-1α and HIF-2α, which, under low-oxygen conditions, escape ubiquitin–proteasome–mediated degradation and translocate into the nucleus to activate a broad set of hypoxia-adaptive and pro-angiogenic genes [18,19]. This HIF-dependent transcriptional response markedly upregulates VEGF-A and other key angiogenic mediators, thereby increasing the dependence of tumor–endothelial interactions on neovascular support [20]. In brain metastases, VEGF-A is highly expressed by tumor cells and further enriched at the tumor–vascular interface, promoting survival, proliferation, and increased permeability of adjacent brain microvascular endothelial cells. These changes not only establish new perfusion routes into the metastatic niche but also form the basis of vasogenic edema. Thus, hypoxia-induced activation of the HIF–VEGF axis is an important driver of sprouting angiogenesis in brain metastases, although its relative contribution varies across vascularization programs and coexists with non-angiogenic mechanisms [21].

### 2.2. VEGF Gradients Orchestrate Tip–Stalk Cell Specification and Endothelial Sprouting

Classical sprouting angiogenesis also occurs in brain metastases and is fundamentally governed by VEGF concentration gradients that direct the functional specialization of brain microvascular endothelial cells [22,23]. In regions of high VEGF concentration, a subset of endothelial cells acquires a tip-cell phenotype characterized by high migratory capacity, abundant filopodia extending toward the hypoxic tumor core, and upregulation of ligands such as DLL4 [22,24,25]. Tip cells secrete large quantities of proteases—particularly matrix metalloproteinases—to locally degrade the vascular basement membrane and extracellular matrix, thereby creating conduits across the brain–tumor interface through which new vascular sprouts can invade the tumor [26,27].

Adjacent endothelial cells, under DLL4–Notch–mediated lateral inhibition, are specified as stalk cells. Upon Notch activation, stalk cells downregulate VEGF-sensitive receptors (e.g., VEGFR2/VEGFR3) and upregulate inhibitory receptors (e.g., VEGFR1), and instead assume responsibilities for ordered proliferation, axial elongation, lumen formation, and maintenance of intercellular junctions [20,24]. As a result, tip cells lead the angiogenic front, while stalk cells elongate and canalize behind them to form new microvascular sprouts that ultimately connect with the dysregulated intratumoral microcirculation, delivering oxygen and nutrients directly into the hypoxic core of the metastatic lesion.

### 2.3. Vascular Maturation and Partial Barrier Reconstruction: The Incomplete Recruitment of Pericytes

Once nascent capillaries extend into the tumor, the next phase is not additional branching but rather stabilization and maturation. Under physiological conditions, endothelial cells—including tumor-associated endothelial cells—secrete PDGF-B/BB to recruit PDGFRβ-positive pericytes and smooth-muscle-like mural cells to the abluminal vessel surface [28]. Tight pericyte–endothelial contact suppresses excessive endothelial proliferation, regulates lumen diameter, reduces leakiness, and supports re-establishment of endothelial junctions and basement-membrane structure, allowing immature, hyperpermeable sprouts to transition into perfusable, relatively stable microvessels [29].

In brain metastases, however, this maturation process is markedly incomplete and spatially heterogeneous. Single-cell transcriptomics and spatial profiling studies show that tumor-associated endothelial cells and mural cells reconstruct not a destroyed BBB but a fundamentally altered blood–tumor barrier (BTB). Within the BTB, the degree of pericyte coverage, astrocytic endfoot support, and retention of critical tight-junction proteins (e.g., claudin-5) varies drastically across regions, producing a highly patchy distribution of vascular permeability [30,31,32].

As a consequence, distinct microenvironments coexist within the same metastatic lesion: (1) Highly permeable, poorly supported abnormal vessels, sparse pericytes, loose endothelial walls, marked leakage, strong contrast extravasation on MRI, extensive vasogenic edema, and a tendency toward microhemorrhage. (2) Vessels retaining BBB-like features have low permeability, restricted drug penetration, and limited immune-cell infiltration [33]. This “hybrid-barrier” nature—neither an intact BBB nor a fully collapsed tumor vasculature—is a defining vascular hallmark of brain metastases. It explains why, within the same patient, some lesions exhibit intense contrast enhancement and substantial edema, whereas others show minimal enhancement, and why metastatic lesions within one individual show highly variable permeability to systemic therapies and immune-mediated responses [34,35,36].

### 2.4. Imaging Phenotypes of Sprouting Angiogenesis

Pathological studies in brain metastases demonstrate that high VEGF expression is accompanied by increased microvascular density, structural vascular abnormalities, disruption of BBB/BTB integrity, incomplete basement membranes, and markedly increased permeability. Radiologically, these microvascular alterations typically manifest as prominent nodular or ring-like contrast enhancement on MRI, often accompanied by extensive surrounding vasogenic edema—features considered characteristic of BTB disruption and active angiogenesis [37,38].

Perfusion MRI studies show that most brain metastases exhibit markedly elevated relative cerebral blood volume (rCBV) and other hemodynamic parameters compared with contralateral normal white matter, and recurrent metastases show substantially higher rCBV than radiation necrosis [38,39,40]. These findings support rCBV as a noninvasive biomarker of tumor angiogenic activity; however, quantitative thresholds and post-processing pipelines remain heterogeneous across studies, limiting cross-platform comparability and motivating prospective validation in brain metastasis–specific cohorts [38,41].

Dynamic contrast-enhanced MRI (DCE-MRI) further demonstrates that, in animal models of lung cancer brain metastasis, K^trans^ values within the tumor and its edematous surroundings significantly exceed those of normal brain tissue and correlate strongly with the penetration of radiolabeled small-molecule tracers—supporting K^trans^ as a quantitative indicator of BTB permeability [42]. Clinical CT perfusion studies also show that elevated permeability–surface area product (PS) in the margin and core of solitary brain metastases correlates positively with VEGF immunohistochemical expression, suggesting that permeability metrics can serve as imaging surrogates of angiogenic activity [38]. In brain metastases, however, perfusion and permeability data that are directly correlated with microvessel density and VEGF expression remain limited to relatively small series, and most mechanistic imaging–pathology correlations derive from glioblastoma cohorts and should be extrapolated cautiously. By analogy to glioblastoma studies and the few available brain metastasis cohorts, current evidence suggests that sprouting angiogenesis in brain metastases may produce locally increased microvascular density and permeability, reflected on imaging as intense enhancement on contrast-enhanced MRI, elevated rCBV on perfusion MRI, and increased K^trans^ or PS values on DCE-MRI or CT perfusion.

To reduce inter-site variability—particularly in trials aiming to operationalize quantitative imaging—BTIP-BM provides consensus recommendations for standardized MRI acquisition in brain metastases, including practical guidance for DSC perfusion imaging [43]. Despite this step forward, prospective brain metastasis–specific cohorts that pre-specify and validate quantitative thresholds for perfusion/permeability-based vascular stratification remain limited [38,41].

Building on these quantitative perfusion/permeability biomarkers, radiomics and deep learning can integrate routine multiparametric imaging into non-invasive signatures that support phenotyping and, potentially, vascular stratification in brain metastases [44]. Multicenter radiomics studies have demonstrated external validation for predicting local control after postoperative stereotactic radiotherapy, and radiomics/ML models can aid differentiation of radiation necrosis from recurrence after SRS—two clinically relevant endpoints for phenotype-guided management [45,46]. For clinical translation, robust feature stability and harmonization across scanners and sites remain essential to ensure reproducibility and generalizability [47].

## 3. Alternative Vascularization Mechanisms and Spatiotemporal Switching of Blood-Supply Modes

**Figure 1:**
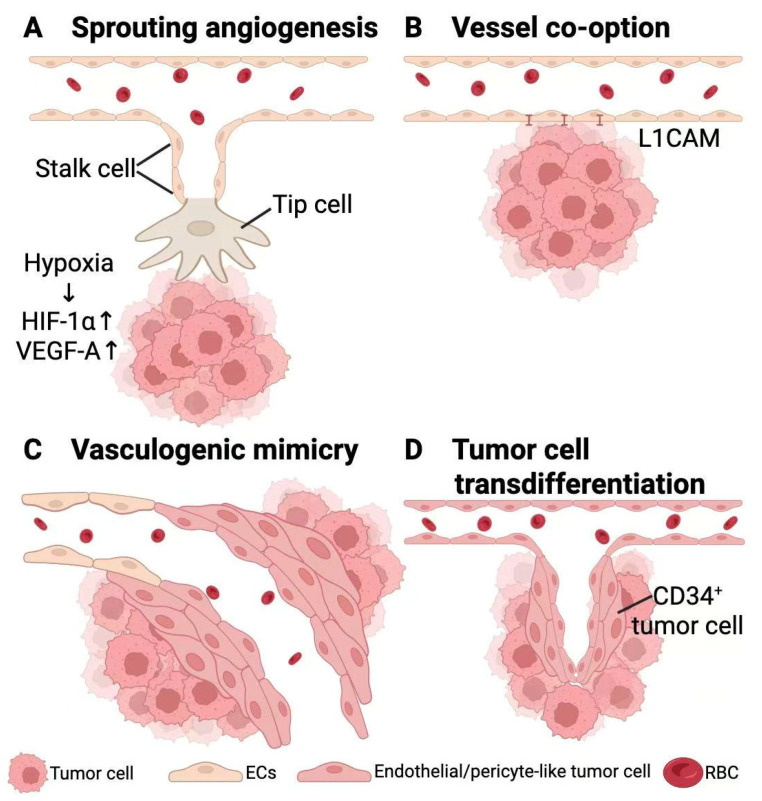
Mechanisms of neovascularization in brain metastases. (**A**) Sprouting angiogenesis. (**B**) Vessel co-option. (**C**) Vasculogenic mimicry. (**D**) Tumor cell transdifferentiation. (Created in BioRender. Xinyun, L. (2026) https://BioRender.com/g27vx6i).

Findings discussed above indicate that VEGF-mediated sprouting angiogenesis remains an important source of perfusion and edema in a substantial proportion of brain metastases. However, this single pathway cannot account for the complex morphology of residual or recurrent lesions after anti-VEGF therapy [48,49]. Increasing evidence now supports that brain metastases exploit multiple vascularization strategies that may operate simultaneously or switch dynamically to sustain growth [50,51]. Beyond classical sprouting angiogenesis, metastatic tumor cells can crawl along pre-existing microvessels and occupy the abluminal surface (vessel co-option), lay down endothelial-like conduits composed of tumor cells themselves (vasculogenic mimicry), or acquire endothelial- or pericyte-like phenotypes and integrate into vessel walls (tumor cell transdifferentiation). These mechanisms are illustrated in Figure 1.

To provide a quantitative clinical anchor for the prevalence of infiltrative tumor–brain interfaces, the MetInfilt trial reported 29/38 (76.3%) infiltrative HGP and 9/38 (23.7%) non-infiltrative HGP [52]. When stratified by primary tumor, lung cancer brain metastases (*n* = 16) displayed a mixed pattern (25% non-infiltrative, 50% epithelial infiltrative, 25% diffuse infiltrative), melanoma metastases (*n* = 6) were predominantly diffuse infiltrative (83.3%), and all evaluable colorectal metastases (*n* = 3) were epithelial infiltrative [52]. Although histological growth patterns are not equivalent to single vascularization modes, an infiltrative (replacement-like) interface is mechanistically compatible with non-angiogenic expansion along pre-existing vessels (including vessel co-option) and perivascular dissemination, motivating interface-aware vascular stratification [53].

### 3.1. Vessel Co-Option

Brain metastases do not universally rely on VEGF-driven sprouting angiogenesis. Many lesions instead hijack the host’s intact microvascular network via vessel co-option (VCO) [54,55]. In this program, metastasis-initiating cells colonize perivascular niches and spread along capillaries through L1CAM-dependent adhesion, enabling early perfusion and expansion without de novo vessel formation—particularly during micrometastatic stages [15,54,56,57].

VCO is an active adhesion–mechanosensing process. L1CAM anchors tumor cells to the microvascular basement membrane via ILK-associated complexes, enabling pericyte-like wrapping and displacement of native pericytes, which remodels the niche and activates mechanotransduction pathways (including YAP) that support outgrowth from dormancy [56,58,59]. Consistent with this concept, the relative stiffness of microvessels compared with neural parenchyma promotes talin-based adhesome–mediated, vascular-guided invasion in brain metastasis models [53]. Clinically, Spanberger et al. identified a VCO-dominant subset with minimal edema, low microvascular density, weak HIF-1α/VEGF signaling, and the lowest maximal rCBV values, yet with the most infiltrative growth and poorest overall survival [60].

Perfusion MRI further supports substantial non-angiogenic perfusion in many brain metastases [38]. In a lung adenocarcinoma model, bevacizumab markedly reduced K^trans^ (and passive tracer permeability) but had minimal effect on steady-state rCBV, indicating restoration of low permeability rather than loss of perfused vessels [42]. Accordingly, VCO-dominant metastases often show modest rCBV elevation, relatively low K^trans^, and limited rCBV response to VEGF blockade—consistent with reliance on intact co-opted microvessels and a comparatively preserved BTB.

To operationalize clinically meaningful subgroups, we propose two end-member phenotypes—angiogenic-dominant lesions and VCO-leaning lesions—while recognizing that mixed or temporally evolving patterns are common [38,54,57]. Angiogenic-dominant metastases typically show prominent enhancement, substantial vasogenic edema, and elevated permeability/perfusion metrics such as K^trans^/PS and rCBV, consistent with leaky neovasculature driven by sprouting angiogenesis [38,61,62]. In contrast, VCO-leaning lesions are expected to present with relatively lower permeability-dominant signals and more infiltrative interfaces, reflecting expansion along pre-existing vessels rather than de novo sprouting [52,54,57]. Because VCO provides a VEGF-independent escape route, VCO-leaning metastases may respond less durably to anti-VEGF monotherapy and may be prioritized for upfront local therapy or investigational VCO-targeted approaches within biomarker-enriched trials [54,55,57]. Practically, subgroup assignment can be initiated using routine MRI plus quantitative perfusion/permeability sequences where available and refined by standardized interface-aware sampling when surgery/biopsy is performed.

Overall, VCO-dominant metastases typically exhibit pronounced microvascular remodeling without significant rCBV elevation, low permeability (low K^trans^), and limited response to VEGF blockade—consistent with their reliance on intact microvessels and relatively preserved BTB integrity.

### 3.2. Vasculogenic Mimicry

Vasculogenic mimicry (VM) denotes matrix-rich, perfusable channels lined by tumor cells rather than endothelial cells and often surrounded by PAS-positive basement membrane–like material [16,63]. In brain metastases, VM has been most consistently documented in melanoma, where higher VM burden correlates with larger lesions and edema; evidence in non-melanoma histologies remains limited and largely derived from small series [16].

VM formation reflects tumor cell plasticity and mechanobiology. Hippo pathway suppression with YAP/TAZ activation promotes VM in melanoma brain metastases, and YAP/TAZ inhibitors (e.g., verteporfin, CA3) suppress VM and reduce intracranial tumor burden in mouse models [63,64]. In TNBC brain metastases, VM has also been linked to ferroptosis tolerance pathways (e.g., PSD4 stabilization), suggesting that VM can act as an adaptive survival program under oxidative and metabolic stress [16,65]. Thus, VM is not merely a morphological curiosity but a survival strategy under oxidative stress and nutrient deprivation—particularly relevant within the hypoxic, ROS-rich brain microenvironment. VM also represents a therapeutic target beyond VEGF signaling.

Although no single imaging feature is pathognomonic, VM-rich melanoma brain metastases often display hemorrhagic components and marked peritumoral edema. In a series of 37 melanoma brain metastases, high VM density was associated with larger tumor size, greater edema, and more pronounced BTB disruption, whereas CD34^+^ vessel density did not correlate with edema or lesion size [16]. Mechanistically, FMOD and SOX2 promote VM via YAP/TAZ activation and correlate with higher edema-to-tumor volume ratios; silencing either gene reduced VM formation in vivo [66]. Collectively, VM contributes a non-endothelial, highly plastic vascular network in MBM and is strongly linked to the imaging phenotype of extensive edema and vascular remodeling.

### 3.3. Tumor Cell Transdifferentiation into Vascular Phenotypes

More extreme vascularization strategy involves direct incorporation of tumor cells into the vessel wall through acquisition of endothelial-like or pericyte-like phenotypes [67]. Two variants exist: tumor cells adopting endothelial-like identity or adopting pericyte-like identity. This mechanism was first rigorously established in glioblastoma, where a subset of CD34+ vascular endothelial cells carry tumor genotypes, indicating neoplastic transdifferentiation. By contrast, in brain metastases, evidence for bona fide tumor-to-endothelial or tumor-to-pericyte transdifferentiation remains largely circumstantial, relying on lineage marker co-expression and spatial proximity rather than genetic lineage tracing.

Emerging evidence suggests similar phenomena in brain metastases. A recent study identified a CD44+ lung cancer brain-metastatic stem-like subpopulation capable of differentiating into pericyte-like cells (“CSC-derived pericytes,” Cd-pericytes). Cd-pericytes adhere tightly to microvessels, remodel the abluminal wall, and exhibit potent transendothelial migration and pro-angiogenic capacity [56,67].

Endothelial-like transdifferentiation may involve Notch- and ERG-driven lineage programs, whereas pericyte-like transdifferentiation may depend on PDGFRβ and TGF-β signaling. However, our understanding remains incomplete, and definitive proof will require integrated genetic lineage-tracing approaches and high-resolution single-cell and spatial transcriptomic analyses in human brain metastases [68]. Transdifferentiation may occur preferentially in select patient subsets (e.g., after radiotherapy or immunotherapy), but defining molecular triggers remains an open challenge.

Because tumor-derived pseudo-endothelial or pseudo-pericyte cells are morphologically and functionally similar to host vascular cells, no distinct radiographic signature exists. These cells may even help maintain local barrier integrity, resulting in regions of low permeability and minimal enhancement on MRI. Current anti-VEGF therapies do not specifically eliminate these transdifferentiated cells. Thus, therapeutically targeting this mechanism may require focusing on PDGFRβ/TGF-β and related differentiation pathways [28].

### 3.4. Dynamic Switching of Vascularization Modes and Therapeutic Implications

Importantly, the three non-classical vascularization strategies described above are not mutually exclusive; instead, brain metastases can switch dynamically between them across time and space. Table 1 summarizes vascularization patterns and potential targets.

Spatially, distinct vascular niches may coexist within a single lesion: (1) The tumor rim often exhibits VEGF-driven angiogenesis with active sprouting, high VEGF/HIF signaling, and dense endothelial buds. (2) The infiltrative front or white-matter-tract–associated regions often display VCO features with high L1CAM, CTNNB1/β-catenin, TGF-β pathway activation, and EMT-related gene expression [30,69].

Temporally, early micrometastatic foci often begin with VCO, maintaining intact BBB/low-permeability BTB and appearing non-enhancing or minimally enhancing on MRI. As lesions enlarge and hypoxia intensifies, VEGF-dependent angiogenesis emerges, producing high-permeability and strongly enhancing regions [57]. With further progression or under anti-VEGF pressure, tumors may reduce reliance on new vessels and shift back toward VCO, VM, or transdifferentiation-based strategies. This plasticity explains why a lesion that strongly enhances with extensive edema may respond transiently to anti-VEGF therapy or radiotherapy, while coexisting non-enhancing, VCO-dominant lesions survive and ultimately drive recurrence.

Clinically, this spatiotemporal heterogeneity reframes the role of anti-vascular therapies in brain metastases: (1) Anti-VEGF agents alleviate edema and improve symptoms but do not eliminate VCO/VM-driven lesions, explaining the frequent disconnect between symptomatic relief and overall survival [16,70]. (2) Imaging features such as enhancement patterns and edema are not neutral descriptors but reflect distinct vascular ecologies, BTB states, immune niches, and drug-accessibility profiles. (3) Future precision strategies must target multiple vascular pathways in parallel—VEGF-dependent sprouts, L1CAM/YAP-driven co-option, and YAP/TAZ- or ferroptosis-related VM pathways—to reduce the “redundancy” of perfusion sources that sustain brain metastases [67,68].

In routine workflows, an imaging-first triage may translate this subgrouping into actionable selection: baseline contrast-enhanced MRI (±DCE/DSC) is used to classify the dominant intracranial lesion as angiogenic-dominant versus VCO-dominant, with mixed cases managed as intermediate phenotypes requiring combination-forward planning [38,52]. For angiogenic-dominant disease, anti-VEGF therapy is prioritized as a backbone to exploit a transient normalization window and facilitate combination with radiotherapy and/or immune checkpoint blockade. Where combination therapy is planned, scheduling RT/ICI during periods of improved perfusion and reduced edema after anti-VEGF initiation is conceptually aligned with normalization-based microenvironment modulation [61,62,71]. For VCO-dominant disease, local therapy (SRS/FSRT or surgery) plus systemic approaches that do not rely on VEGF blockade are emphasized, while anti-VEGF is reserved for edema control or as part of carefully selected combinations supported by emerging brain-metastasis trials [72,73].

## 4. Therapeutic Strategies Targeting Angiogenesis in Brain Metastases

### 4.1. Mechanistic Rationale of Anti-Angiogenic Therapy: Vascular Normalization and BTB Modulation in Brain Metastases

The fundamental rationale for anti-angiogenic therapy (AAT) in brain metastases is to suppress VEGF/VEGFR-driven angiogenic signaling and induce a transient state of vascular normalization, thereby disrupting the pathological cycle of abnormal vasculature, elevated interstitial pressure, poor perfusion, and profound hypoxia. Through this temporary normalization process, AAT lowers peritumoral edema and interstitial pressure, enhances perfusion and the delivery of chemotherapeutic agents or targeted therapies, and improves radiosensitivity and immune-cell infiltration [62,74,75]. These effects are particularly meaningful in the setting of brain metastases, where the formation of a highly heterogeneous BTB restricts the penetration of systemic therapies. A brief window of normalized vasculature may therefore improve transendothelial trafficking of drugs and immune effector cells. Consequently, in clinical practice, AAT functions more as a microenvironmental modulator that creates conditions favorable for combination therapy, rather than as a standalone intervention with robust direct anti-tumor efficacy [76,77].

To capitalize on the anti-VEGF vascular-normalization window and mitigate BTB-limited exposure, BTB-disruptive/delivery-enabling interventions can be time-locked as adjuncts to systemic therapy [61,78]. MR-guided low-intensity focused ultrasound (FUS) with microbubbles can reversibly open the BBB/BTB locally, and recent clinical-trial syntheses support feasibility with generally favorable safety when acoustic parameters are controlled [79,80,81]. A nanomodulator strategy co-targeting endothelial WNT signaling and pericyte programs has been shown to reversibly open the BTB and increase intracranial drug accumulation with therapeutic benefit in a breast cancer brain metastasis model [12]. Magnetic field–responsive nanocarriers offer a complementary physical targeting layer by improving intracranial retention/targeting of superparamagnetic constructs and potentially reducing systemic exposure, although evidence remains largely preclinical [82,83]. Conceptually, aligning these modalities with the normalization window—when edema and interstitial pressure fall and perfusion improves—may maximize intratumoral exposure while limiting off-target neurotoxicity [61,84]. Finally, because VEGF-independent escape (vessel co-option, vasculogenic mimicry) is linked to mechanotransduction and Hippo/YAP–TAZ programs, combining delivery enhancement with Hippo-pathway targeting warrants evaluation to improve penetration while constraining adaptive vascular plasticity [16,85,86].

### 4.2. Monotherapy with Anti-VEGF Agents

Bevacizumab, a recombinant humanized neutralizing antibody targeting VEGF-A, rapidly reduces vascular permeability and vasogenic edema and enhances perfusion and drug delivery during the transient “vascular normalization” window. These biological effects underpin its value in brain metastatic disease [61,74]. Increasing clinical evidence and contemporary guidelines consistently indicate that bevacizumab is an effective therapeutic option for radiation necrosis (RN) and steroid-refractory peritumoral edema [87,88]. Across retrospective cohorts, prospective small-scale studies, and real-world experiences, bevacizumab repeatedly induces radiographic edema regression, facilitates steroid tapering, and improves neurological function. In practice, the most commonly adopted regimens involve doses of 5–10 mg/kg every 2–3 weeks for 4–6 cycles, though both high- and low-dose strategies have been explored. Edema recurrence following drug discontinuation is not uncommon, and individualized maintenance or re-challenge approaches may achieve renewed symptomatic improvement [89,90]. Ongoing prospective randomized trials such as A221208/BeSt will further clarify the standardized role of bevacizumab monotherapy in RN.

When bevacizumab is used with anti-tumor intent, unselected patient populations typically experience radiographic or symptomatic improvement without consistent overall-survival benefit, a key reason why current guidelines remain cautious [87]. Some studies suggest potential benefit in highly angiogenesis-dependent subsets, such as colorectal cancer brain metastases, but small sample sizes, treatment heterogeneity, and concomitant local therapies limit generalizability [91]. Taken together, the most robust clinical consensus is that bevacizumab monotherapy primarily serves as a symptom-directed intervention for RN and refractory edema [88]. When direct tumor control is the goal, bevacizumab is best contextualized within a combinatorial framework in which tumor angiogenic dependency is evaluated using imaging or molecular phenotyping. Safety considerations remain essential, as anti-VEGF therapies may cause hypertension, proteinuria, hemorrhage, thromboembolic events, impaired wound healing, and rare complications such as PRES [92,93]. Meta-analytic evidence suggests no significant increase in severe intracranial hemorrhage compared with non-AAT controls, although heterogeneity across tumor types and concomitant treatments requires individualized assessment [94,95].

### 4.3. Anti-VEGF Therapy Combined with Immune Checkpoint Inhibitors

Anti-angiogenic therapy can improve perfusion and oxygenation, reduce hypoxia, normalize abnormal vascular permeability, and attenuate the immunosuppressive microenvironment, thereby enhancing antigen presentation and CD8^+^ T-cell infiltration [62,71]. These changes provide a strong biological foundation for combining AAT with immune checkpoint inhibitors (ICIs). In melanoma brain metastases (MBM), particularly in patients without prior intracranial local therapy and without neurological symptoms or hemorrhage risk, dual immune checkpoint blockade such as nivolumab plus ipilimumab has demonstrated impressive intracranial activity. The asymptomatic cohort of CheckMate-204 reported an intracranial objective response rate (ORR) of approximately 55%, with durable long-term control, and the Australian ABC trial similarly showed superior intracranial response and prolonged survival with dual ICIs compared with monotherapy, with sustained benefit across seven-year follow-up [96,97,98].

Beyond these immunotherapy-only regimens, the combination of AAT with ICI has shown promising results in prospective trials. A rigorously designed two-center phase II study evaluating pembrolizumab plus bevacizumab in untreated, asymptomatic MBM (5–20 mm, steroid-free, and without hemorrhage) reported an intracranial response rate of 54.1% and a median OS of 4.3 years, with manageable toxicity. Although the single-arm design precludes definitive causal inference, these data provide strong support for the hypothesis that VEGF blockade may augment intracranial benefit from PD-1 inhibition in melanoma brain metastases [73]. Taken together with other early-phase studies, they highlight AAT–ICI combinations as a particularly promising strategy in melanoma brain metastases, albeit one that remains supported predominantly by phase II, single-arm data.

In non-melanoma histologies, atezolizumab plus bevacizumab with platinum doublet chemotherapy improved OS and PFS in metastatic nonsquamous NSCLC (IMpower150), with exploratory analyses suggesting consistent benefit in patients with stable baseline brain metastases [99]. By contrast, the landmark IMbrave150 trial in unresectable hepatocellular carcinoma demonstrated systemic survival benefit from atezolizumab–bevacizumab but included virtually no patients with active brain disease [100]. Thus, while the IMpower150 and IMbrave150 experiences mainly reinforce the systemic efficacy and feasibility of PD-(L)1–VEGF strategies rather than providing definitive intracranial efficacy signals, multiple phase III randomized trials in extracranial solid tumors offer higher-level evidence on dosing, treatment duration, and safety profiles that can inform rational AAT–ICI development for brain metastases [101].

In metastatic renal cell carcinoma, KEYNOTE-426 combined pembrolizumab (200 mg every 3 weeks) with axitinib (5 mg twice daily), continued until disease progression or unacceptable toxicity, and demonstrated superior survival outcomes versus sunitinib [102]. CheckMate 9ER paired nivolumab (240 mg every 2 weeks) with cabozantinib (40 mg once daily), with nivolumab administered for up to 2 years, and also improved OS and PFS versus sunitinib in a phase III setting [103]. The CLEAR trial further established pembrolizumab (200 mg every 3 weeks) plus lenvatinib (20 mg once daily) as an effective VEGFR-targeted ICI combination with significant survival benefit compared with sunitinib [104].

As potential biomarkers, PD-L1 status can inform baseline ICI sensitivity in selected tumor contexts, yet clinically meaningful discordance between primary tumors and brain metastases has been reported, supporting intracranial reassessment when feasible for phenotype-guided combination strategies [105]. Tumor mutational burden (TMB) has shown tumor-type–dependent associations with ICI response, but pan-cancer analyses caution against treating TMB as a universally reliable predictor, so its use for selecting AAT–ICI combinations in BM should remain tumor-contextual and hypothesis-generating [106]. In parallel, transcriptomic signatures capturing angiogenesis and T-effector programs have been associated with differential outcomes to atezolizumab–bevacizumab versus sunitinib in randomized RCC datasets, providing a framework for future BM-specific validation of vascular–immune stratification [107].

### 4.4. Anti-VEGF Therapy Combined with Radiotherapy

Leveraging the vascular-normalization window described in Section 4.1, AAT has been explored as an adjunct to radiotherapy by transiently improving perfusion/oxygenation and reducing edema, potentially enhancing the therapeutic ratio of SRS/HSRT. In brain metastases, clinical data are largely limited to improvements in edema control and potentially lower rates of symptomatic radiation necrosis, whereas a consistent survival advantage has yet to be demonstrated [108]. Outside the BM population, NRG Oncology/RTOG 1205 randomized bevacizumab-naïve recurrent glioblastoma to bevacizumab alone versus bevacizumab (10 mg/kg every 2 weeks) with re-irradiation (35 Gy in 10 fractions), providing randomized evidence on feasibility and scheduling of VEGF blockade with CNS radiotherapy. The combined arm improved progression-free survival without a statistically significant overall-survival benefit and showed acceptable toxicity, illustrating that radiobiologic synergy and edema control do not necessarily translate into consistent OS gains [109]. Tumor perfusion and oxygenation have been correlated with SRS local control, and early quantitative DCE/DSC-MRI or diffusion-imaging biomarkers can predict mid-treatment outcomes, lending imaging-based support to a strategy in which vascular normalization precedes radiation delivery [110,111,112].

In the context of radiation necrosis, a 2024 systematic review from the International Stereotactic Radiosurgery Society (ISRS), summarizing 28 studies and 1125 patients, reported a weighted symptomatic improvement rate of approximately 86%, edema reduction in about 74% of cases, contrast-enhancing volume reduction in 88%, and steroid discontinuation in roughly 45% [88]. Low-dose bevacizumab exhibited symptomatic benefits comparable to or potentially greater than higher-dose regimens, underscoring that anti-edematous and anti-inflammatory effects may represent key mechanisms. For peri-radiation applications, multiple prospective and retrospective cohorts indicate that concurrent bevacizumab with SRS/HSRT reduces edema, limits necrosis-like radiographic progression, and improves neurological outcomes; doses of ≤7.5 mg/kg administered every three weeks appear effective and safe [113].

### 4.5. Anti-VEGF Therapy Combined with Targeted Therapy

In patients with driver-mutated NSCLC, where CNS relapse is a major concern, highly CNS-penetrant small-molecule TKIs—most notably the third-generation EGFR inhibitor osimertinib—form the cornerstone of intracranial disease control. Randomized trials and dedicated CNS subgroup analyses consistently report strong intracranial responses and prolonged CNS-PFS/OS, establishing osimertinib as the first-line standard of care [114,115]. Based on systemic disease models, AAT may complement TKIs by modulating the abnormal vascular microenvironment during the normalization window and potentially improving drug delivery and edema control [116].

Earlier attempts to combine first-generation EGFR-TKIs with bevacizumab demonstrated improved PFS but not OS. The trials NEJ026 and BEVERLY were conducted in patients with advanced EGFR-mutant NSCLC. Although stable, treated brain metastases were allowed in some of these trials, intracranial outcomes were not primary endpoints, and dedicated CNS subgroup analyses remain limited. The randomized phase III trial NEJ026 (erlotinib + bevacizumab versus erlotinib alone) showed significant PFS prolongation with the combination but no survival benefit, accompanied by higher rates of proteinuria and hypertension [117]. The Italian BEVERLY study produced similar findings, reinforcing the notion that such combinations may delay progression without improving long-term survival [118]. In the contemporary osimertinib-first era, randomized trials have not demonstrated benefit from adding bevacizumab: the WJOG9717L trial and its final analysis showed no improvement in PFS or OS and increased toxicity, with biomarker exploration failing to identify a consistent benefit subgroup. Meta-analyses confirm these results, indicating that routine addition of AAT to osimertinib is unsupported [119]. For renal cell carcinoma brain metastases, multicenter retrospective analyses indicate that cabozantinib may have meaningful intracranial activity with acceptable toxicity, though prospective validation is required. For ALK-positive NSCLC, long-term follow-up of authoritative randomized trials strongly endorses the use of second- or third-generation ALK inhibitors with high CNS penetration, such as alectinib or lorlatinib, while AAT does not form part of standard first-line strategies [120].

Taken together, intracranial management of driver-mutated NSCLC should primarily rely on CNS-penetrant TKIs. Current evidence supports using anti-angiogenic therapy primarily for symptomatic indications such as severe edema or for specific transitional scenarios, such as bridging radiotherapy or managing partial resistance, ideally within clinical-trial settings. Future research should incorporate intracranial-specific endpoints, integrate imaging and molecular markers of vascular dependency, perfusion/oxygenation, and immune infiltration, and match therapeutic timing to the vascular-normalization window to rigorously determine whether AAT+TKI combinations can yield survival benefit in molecularly defined patient subsets.

Across prospective studies, evidence has accumulated from radiosensitization in local therapies, treatment of salvage brain metastases and radiation necrosis, and combination strategies with EGFR-TKIs or ICIs. Collectively, these data outline the early but expanding evidence landscape of anti-VEGF strategies in brain metastasis. For clarity, the major clinical trials evaluating AAT in this setting are summarized in Table 2.

## 5. Mechanisms of Resistance and Adaptation to Anti-Angiogenic Therapy

### 5.1. Mechanisms of Resistance to Anti-Angiogenic Therapy in Brain Metastases

In clinical practice, AAT in brain metastases is characterized by meaningful benefit in a subset of patients, yet with limited durability. This pattern strongly suggests that both intrinsic resistance and treatment-induced adaptive escape are prevalent and highly heterogeneous. It also explains why rapid radiographic improvement of enhancement or edema does not consistently translate into measurable overall survival (OS) gains [51]. Mechanistically, brain metastases can maintain perfusion despite inhibition of the VEGF axis by engaging multiple non-canonical vascularization programs. VCO, VM, and the acquisition of pericyte-like or endothelial-like phenotypes by tumor cells are increasingly recognized as plausible alternative vascular programs, particularly in preclinical models in which VEGF-dependent sprouting angiogenesis is suppressed. Observational studies of human brain metastases suggest that such non-angiogenic patterns may contribute to primary or acquired resistance to anti-angiogenic therapy, but definitive causal evidence remains limited [54]. These processes have been discussed in detail earlier in this review. At the same time, the BTB exhibits pronounced spatiotemporal heterogeneity and a hybrid state in which areas of abnormal permeability coexist with regions that retain partial barrier function. This results in striking variations in drug exposure and therapeutic response among distinct intracranial lesions within the same patient. Functional imaging markers of perfusion and permeability, including K^trans^ on DCE-MRI and DSC-derived hemodynamic parameters, correlate with early intracranial response and provide a pharmacokinetic framework for understanding the dissociation often observed between CNS-PFS and OS [121,122,123].

At the microenvironmental level, sustained hypoxia and dysfunctional perfusion perpetuate an immunosuppressive milieu and recruit pro-angiogenic myeloid cells, including Tie2^+^ tumor-associated macrophages. Although appropriately timed and dosed AAT may transiently improve perfusion and enhance T-cell infiltration, the so-called “normalization window” is brief and subject to wide inter-individual variability [71,74,75]. This variability likely contributes to the inconsistent reproducibility of AAT-mediated radiosensitization and immunotherapy enhancement. Additional modifiers—including corticosteroid exposure, tumor PD-L1 expression, and host immune competence—further influence the therapeutic impact of AAT combined with immune checkpoint inhibition, leading to highly variable clinical benefit across patients.

### 5.2. Strategies to Overcome Resistance to Anti-Angiogenic Therapy

Given the resistance mechanisms described above, an increasingly emphasized direction is to rationalize AAT-based combinations and timing based on lesion-level vascular biology, rather than applying AAT uniformly [124]. To translate phenotype-guided therapy into routine workflows, a minimal three-step process can be embedded into multidisciplinary brain-metastasis care pathways recommended by contemporary guidelines [87,125]. First, standardized baseline and follow-up MRI acquisition (3D post-contrast T1 with optional DSC/DCE metrics) should follow consensus imaging protocols to maximize comparability across timepoints and centers [43]. Second, lesion-level phenotyping can integrate enhancement/edema patterns with quantitative surrogates of vascularity and permeability (e.g., rCBV, K^trans^) and, when available, amino-acid PET to estimate whether a lesion is more angiogenic/permeable versus co-option-leaning and relatively barrier-preserved [126,127]. Third, this phenotype estimate can guide therapy selection and timing—deploying AAT primarily as a delivery- and synergy-enabling microenvironmental modulator with RT/ICI in angiogenic-dominant lesions, while prioritizing local therapy and/or non-VEGF targets in lesions suspected to rely on vessel co-option or vasculogenic mimicry [15,124]. Given the intra-patient heterogeneity and treatment-driven vascular plasticity, re-stratification at each major decision point using harmonized imaging timepoints and standardized response-assessment frameworks is essential [128].

The synergy between AAT and immune checkpoint inhibitors is grounded in a biologically coherent sequence in which vascular normalization improves perfusion and oxygenation, alleviates immunosuppression, and enhances T-cell infiltration [129]. This mechanistic foundation has translated into encouraging clinical signals: in immunotherapy-sensitive entities such as melanoma brain metastases, combinations of anti-VEGF agents with PD-1 inhibitors have achieved substantial intracranial response rates and prolonged survival in phase II cohorts (e.g., pembrolizumab plus bevacizumab), although the absence of randomized comparisons against checkpoint blockade alone limits causal inference [73]. Dual checkpoint blockade regimens such as nivolumab plus ipilimumab also provide high and durable intracranial response rates in untreated, asymptomatic melanoma brain metastases, and serve as an important benchmark for evaluating AAT–ICI combinations [96,97].

In the radiation therapy domain, the strategy of administering AAT prior to radiotherapy is being explored to enhance radiosensitivity and reduce the risk of radiation necrosis [130]. Clinical data discussed previously support the premise that vascular normalization can improve local control by optimizing the physiological conditions under which radiation is delivered.

Beyond combination strategies with immunotherapy or radiotherapy, increasing attention is being directed toward targeting alternative vascularization pathways directly. For vessel co-option–dominant phenotypes, disrupting the L1CAM–YAP axis or inhibiting mechanotransduction pathways may offer therapeutic leverage. For vasculogenic mimicry, suppression of YAP/TAZ activity or modulation of metabolic adaptations such as ferroptosis sensitivity may be effective. In tumors that rely on transdifferentiation of cancer cells into endothelial-like or pericyte-like phenotypes, blockade of lineage-specifying signals—such as PDGFRβ, Notch, or TGF-β—may be required to impair this adaptive program [131,132]. Most of these strategies remain at the preclinical or early clinical investigational stage, but they represent important future directions in the development of multi-axis vascular-targeted therapy for brain metastases.

## 6. Conclusions and Future Directions

The vascular ecosystem of brain metastases is defined by a malignant cycle of abnormal tumor vasculature, elevated interstitial pressure, impaired perfusion, and profound hypoxia, superimposed upon the unique spatiotemporal heterogeneity of the brain’s blood–tumor barrier [79,133]. This architecture not only supports metastatic progression but also shapes intracranial drug exposure, governs the effectiveness of radiotherapy, and dictates the trajectory of therapeutic resistance. Beyond classical sprouting angiogenesis, metastatic lesions draw on multiple alternative vascularization programs—including vessel co-option, vasculogenic mimicry, and tumor-cell acquisition of endothelial- or pericyte-like phenotypes—and dynamically shift among these routes to withstand therapeutic pressure [96]. These observations underscore the need for management strategies that address the full spectrum of vascular redundancies rather than focusing solely on VEGF-dependent angiogenesis.

From a therapeutic perspective, anti-angiogenic therapy has established symptomatic benefits in brain metastases, notably through reducing peritumoral edema, alleviating neurological deficits, and treating radiation necrosis [51,134]. However, because tumors can exploit non-angiogenic blood-supply pathways, monotherapy directed at VEGF rarely achieves durable control, and adaptive resistance is nearly universal. Combination strategies have therefore become increasingly prominent [135]. The integration of anti-angiogenic agents with immunotherapy, radiotherapy, or targeted therapy leverages complementary mechanisms and has already shown encouraging improvements in intracranial control in selected subtypes such as melanoma and non-small-cell lung cancer, mainly in phase II and single-arm studies [134]. Further randomized, brain-metastasis-focused trials are needed to refine the optimal therapeutic context for each combination and to delineate subtype-specific indications [13,119].

Radiomics and deep-learning approaches are increasingly used to translate routine clinical imaging into quantitative, high-dimensional descriptors of lesion biology and the peritumoral microenvironment, providing a scalable layer of non-invasive phenotyping in brain metastases [44,136]. Recent multicenter studies have shown that radiomics features extracted from the enhancing lesion or resection cavity (T1-CE) and surrounding edema (T2-FLAIR) can improve prediction of local control after stereotactic radiotherapy beyond clinical variables alone, underscoring potential value for outcome stratification [46,137]. Radiomics and machine learning have also demonstrated promise for distinguishing radiation necrosis from tumor progression after radiosurgery, facilitating more accurate response assessment when conventional imaging is equivocal [45]. Beyond clinical endpoints, imaging-derived models have been associated with both the likely cancer of origin and selected molecular features (e.g., EGFR mutational status in NSCLC brain metastases), supporting radiogenomic enrichment strategies for personalized therapy [138]. Within a vascular-ecosystem framework, integrating radiomic signatures of enhancement heterogeneity, perfusion/permeability distributions, and peritumoral texture with mechanistic knowledge of angiogenic versus vessel co-option programs could—if prospectively validated against histopathology and molecular profiling—enable hypothesis-generating vascular stratification and guide rational selection of AAT-based combinations [15,44]. Achieving clinical-grade deployment will require rigorous harmonization and external validation across scanners, institutions, and therapeutic contexts to ensure robustness and trustworthiness [47,139]. Such “virtual biopsies,” if prospectively validated in brain metastasis–specific cohorts and standardized through harmonized acquisition frameworks (e.g., BTIP-BM), could ultimately guide individualized treatment selection and move brain metastasis management closer to true precision oncology [43].

Complementing this imaging- and radiomics-driven framework, omics-based profiling—particularly single-cell and spatially resolved approaches—has begun to delineate the cellular and molecular architecture of human brain metastases, including vascular and perivascular compartments that are not captured by bulk assays alone [30,140]. Spatial transcriptomics and proteomics are uncovering regional heterogeneity and therapy-resistant microenvironments at the tumor–stroma interface, providing a molecular basis for why vascular phenotypes and treatment responses vary across lesions [141]. Moreover, single-cell multi-omics approaches are identifying candidate biomarkers and therapeutic targets related to BTB modulation, immune suppression, and metabolic rewiring [142,143]. When integrated with AI-driven analytics, these datasets may enable predictive modeling of vascular phenotypes and treatment sensitivity at the individual lesion level [144,145].

In summary, advances in the understanding of neovascularization in brain metastases are reshaping our perspective on their biology and therapeutic vulnerabilities [125]. As vascular-ecosystem–based stratification frameworks mature and as novel combination approaches continue to evolve, more refined and clinically testable treatment algorithms are likely to emerge [43,87]. The integration of radiomics with molecular profiling and rigorously designed clinical trials will be central to developing vascular-targeted strategies that not only control symptoms but also meaningfully extend survival and improve the quality of life for patients with brain metastases. Achieving these goals will require interdisciplinary collaboration to translate mechanistic insights into clinically actionable interventions capable of delivering durable benefit [52]. By explicitly linking vascular mechanisms, imaging phenotypes, and therapeutic strategies within a single conceptual framework, this review aims to provide a foundation for future brain-metastasis trials that are designed and interpreted through the lens of the vascular ecosystem.

## Figures and Tables

**Table 1:** Key signaling pathways, imaging/pathological features, and potential therapeutic strategies across distinct vascularization modes in brain metastases.

Blood-Supply Pattern	Key Pathways/Molecules	Imaging/Pathological Clues	BTB Accessibility	Candidate Drugs/Strategies	Optimal Combinations and Timing	Applicable Populations/Clinical Scenarios	Major Pitfalls
**Sprouting angiogenesis**	HIF-1/2α–VEGF-A; DLL4–Notch; PDGF-B/PDGFRβ; ANGPT–TIE2 axis	Marked contrast enhancement, ↑rCBV, ↑K^trans^; histology shows high microvascular density and immature pericyte coverage	Moderate–high, with pronounced spatial heterogeneity	Bevacizumab; VEGFR tyrosine kinase inhibitors; agents targeting the ANGPT/TIE2 axis	Anti-angiogenic therapy to induce a “vascular normalization” window followed by SRS/HSRT; in selected patients, concurrent ICI during the normalization window	Lesions with strong enhancement, elevated rCBV, and prominent peritumoral edema; patients at high risk for, or already experiencing, radiation necrosis or severe edema	VEGF inhibition may promote a shift toward alternative blood supplies such as vessel co-option or VM; radiographic response does not necessarily translate into OS benefit
**Vessel co-option**	L1CAM–integrin β1–ILK–YAP/TAZ mechanotransduction axis	Often absent or only mild enhancement in early stages; tumor cells track along pre-existing microvessels; histology shows low VEGF expression and L1CAM positivity	Low–moderate; BBB/BTB relatively preserved in most regions	Inhibitors of adhesion and mechanotransduction pathways (e.g., L1CAM, ILK, YAP/TAZ; currently largely preclinical)	Local control primarily with RT; cautious ICI combination when steroid dose is low and lesion volume is limited	Micrometastases, low-perfusion lesions, or residual/recurrent disease after prior VEGF-targeted therapy	Lack of standardized imaging or molecular markers; easily confused with sprouting angiogenesis on imaging, making precise stratification difficult
**Vasculogenic mimicry (VM)**	VE-cadherin–EphA2–MMP axis; YAP/TAZ; FMOD–SOX2 and metabolism/ferroptosis-related pathways	PAS^+^/CD31^−^ vessel-like channels; highly heterogeneous perfusion; frequently associated with intratumoral hemorrhage and extensive edema (particularly common in MBM)	Generally low; some regions contiguous with necrotic/severely hypoxic areas	VM formation inhibitors; combined approaches targeting metabolism and ferroptosis sensitivity (mostly exploratory)	Timing of combination with RT or ICI guided by hypoxia/VM biomarkers	Repeatedly recurrent, hypoxia- and necrosis-rich brain metastases, such as subsets of MBM and TNBC brain metastases	Diagnosis requires a multi-evidence chain (e.g., PAS/CD31 staining); limited extrapolation across tumor types; current clinical implementability remains low
**Tumor cell transdifferentiation into endothelial-like cells**	Notch–ERG and other endothelial lineage programs; acquired VEGFR2 expression	CD31/CD34^+^ “endothelial-like” cells carrying tumor genotypes; contribution to abnormal vessel walls	Low–moderate, depending on the extent of tumor-derived endothelial coverage	Agents blocking endothelial lineage programs (e.g., Notch or VEGFR2 inhibitors; evidence largely preclinical)	Personalized combination with RT or ICI based on molecular profiling; no standardized regimen yet	Molecular subgroups harboring tumor-derived endothelial-like cells (requires confirmation by pathology or single-cell sequencing)	Lineage evidence remains limited; difficult to distinguish from reactive endothelium; lack of mature classification systems and targeted agents
**Tumor cell transdifferentiation into pericyte-like cells**	PDGFRβ, NG2, TGF-β signaling; pericyte-lineage–associated programs	Co-expression of PDGFRβ, NG2 and tumor genotypes; irregular thickening of vessel walls or abnormal pericyte proliferation	Intermediate; permeability similar to regions with reactive pericytes	PDGFRβ inhibitors or multi-target TKIs; combined with anti-angiogenic therapy (AAT) and RT to modulate edema and vascular stability	Initial use of AAT to reduce permeability and edema, followed by RT; in some cases, maintenance therapy to control edema	Patients with edema-dominant presentations where pericyte abnormalities are suspected; pericyte-enriched lesions	Difficult to distinguish tumor-associated pericytes from normal/reactive pericytes; broad PDGFR inhibition may disrupt normal vascular homeostasis

Most candidate drugs and strategies listed here—particularly those targeting YAP/TAZ, VM-related pathways, or tumor-cell transdifferentiation—are currently supported mainly by preclinical data or early-phase clinical observations and should be regarded as exploratory rather than established standards of care.

**Table 2:** Summary of prospective clinical trials evaluating anti-angiogenic strategies in brain metastases or in advanced cancer populations including patients with brain metastases.

ClinicalTrials.gov Identifier	Study Design	Intervention	Study Population	Primary Endpoint(s)	Sample Size	Key Results	Safety
NCT01332929 (REBECA)	Phase I, single-arm, dose-escalation	Bevacizumab + whole-brain radiotherapy (WBRT 30 Gy in 10 fractions)	Brain metastases from solid tumors eligible for WBRT	Dose-limiting toxicity (DLT), recommended dose; intracranial objective response rate (ORR)	*n* = 19	No DLTs were observed. The recommended regimen was bevacizumab 15 mg/kg q2w + WBRT. Approximately half of the patients achieved intracranial response at 3 months, suggesting that bevacizumab may potentiate radiotherapy and improve edema control.	Toxicities were manageable, mainly bevacizumab-associated hypertension and proteinuria; no signal of increased symptomatic intracranial hemorrhage.
NCT00800202 (BRAIN)	Phase II, open-label, multi-cohort	Bevacizumab + carboplatin/paclitaxel (treatment-naïve) or + erlotinib (post-systemic therapy)	Non-squamous NSCLC with untreated, asymptomatic brain metastases	ORR and PFS per predefined regimens; pre-specified intracranial efficacy analysis	*n* = 91	In NSCLC patients with untreated brain metastases, both overall and intracranial ORR were ~60%, with median PFS of ~6–7 months, demonstrating meaningful intracranial activity of bevacizumab-based systemic therapy in selected patients.	Safety profile consistent with prior bevacizumab + chemotherapy experience (hypertension, proteinuria, myelosuppression); serious intracranial bleeding was rare.
NCT01281696 (BEEP)	Phase II, open-label, single-arm	Bevacizumab + etoposide + cisplatin (WBRT allowed in some cases)	Heavily pretreated brain metastases/meningeal disease, predominantly from breast cancer	Intracranial ORR	*n* ≈ 20	Reported intracranial ORR was ~70–80%, with median intracranial PFS ~7 months, indicating preserved activity of the “BEEP” regimen even in pretreated brain metastases.	Grade 3–4 myelosuppression was common; bevacizumab-related hypertension/proteinuria occurred but were manageable. Severe intracranial hemorrhage was rare.
NCT01898130	Phase II, open-label, single-arm	Bevacizumab monotherapy (10 mg/kg q2w)	Recurrent or progressive solid-tumor brain metastases after radiotherapy	Radiographic response rate; 6-month PFS (PFS6)	*n* = 27	Intracranial response rate ~60%; PFS6 ~45%; median PFS ~5 months, median OS ~9–10 months, indicating symptomatic and radiographic benefit in the salvage setting.	Generally well tolerated; mostly grade 1–2 hypertension, proteinuria, lymphopenia; occasional thrombotic events; no unexpected severe intracranial hemorrhage.
NCT02490878 (A221208/BEST)	Phase II, randomized, double-blind	Bevacizumab vs. placebo + standard corticosteroids	Imaging-confirmed radiation necrosis after surgery or radiotherapy (brain metastases or primary brain tumors)	Change in necrosis volume, steroid tapering success, neurological improvement	*n* ≈ 110	Compared with placebo, bevacizumab led to significantly greater reductions in necrosis volume, higher steroid-taper success, and improved neurological function and quality of life, establishing a strong evidence base for bevacizumab in radiation necrosis.	Increased bevacizumab-associated AEs (hypertension, proteinuria, venous thrombosis), mostly grade 1–2; no excess intracranial hemorrhage.
NCT02681549	Phase II, open-label, single-arm	Pembrolizumab + bevacizumab	Untreated melanoma or non-squamous NSCLC brain metastases; PD-1/PD-L1 inhibitor–naïve	Intracranial ORR	*n* ≈ 60	In untreated MBM, the combination achieved intracranial ORR >50% with durable responses; several patients attained long-term intracranial control and discontinued steroids, supporting “vascular normalization + immune potentiation.”	Overall well tolerated; immune-related AEs and bevacizumab-associated hypertension/proteinuria were mostly grade 1–2; no signal of treatment-related major intracranial hemorrhage.
NCT02366143 (IMpower150)	Phase III, randomized, three-arm	Atezolizumab + bevacizumab + carboplatin/paclitaxel (ABCP) vs. atezolizumab + carboplatin/paclitaxel vs. bevacizumab + carboplatin/paclitaxel (BCP)	Chemotherapy-naïve advanced non-squamous NSCLC; allowed previously treated, stable brain metastases	OS, PFS; exploratory analysis of time to new brain metastases	*n* = 1202	ABCP significantly prolonged OS and PFS in the overall population. Exploratory subgroup analyses suggested benefit trends in patients with prior EGFR-TKI therapy and in those with baseline brain/liver metastases. Time to new brain metastases was numerically delayed, but CIs were wide.	Safety consistent with the ABCP regimen (myelosuppression, hypertension, proteinuria); no increase in CNS-specific toxicity in patients with brain metastases.
NEJ026 (UMIN000017069)	Phase III, randomized, open-label	Erlotinib + bevacizumab vs. erlotinib	First-line EGFR-mutated advanced non-squamous NSCLC; allowed stable brain metastases after local therapy	PFS	*n* = 228	Combination therapy significantly prolonged PFS (16.9 vs. 13.3 months). However, active brain metastases were largely excluded and no intracranial endpoints were predefined; conclusions regarding intracranial efficacy remain indirect.	Increased grade 3–4 hypertension, proteinuria, rash in the combination arm; most AEs manageable; no signal of excess hemorrhagic stroke.
WJOG9717L (UMIN000030206)	Phase II, randomized, multicenter	Osimertinib + bevacizumab vs. osimertinib	First-line EGFR-mutated NSCLC; allowed stable, asymptomatic brain metastases after local therapy	PFS	*n* ≈ 120	The combination did not significantly improve PFS over osimertinib monotherapy. Some intracranial exploratory endpoints showed non-significant favorable trends, but the trial was underpowered for CNS outcomes.	Adding bevacizumab increased VEGF-related toxicities (hypertension, proteinuria), but overall safety remained acceptable; no new safety signals.
BEVERLY (EUDRACT 2010-020519-28)	Phase III, randomized, multicenter	Erlotinib + bevacizumab vs. erlotinib	First-line EGFR-mutated NSCLC; baseline brain metastases excluded	PFS	*n* ≈ 160	No significant improvement in PFS or OS with the combination. Because brain metastases were excluded, the study provides only indirect insight into whether improved systemic control reduces the subsequent risk of CNS involvement.	Increased bevacizumab-related hypertension and proteinuria, but generally manageable. Exclusion of brain metastases prevents assessment of intracranial safety.

## Data Availability

No new data were created or analyzed in this study.

## Abbreviations

The following abbreviations are used in this manuscript:

AAT	Anti-angiogenic therapy
AE/AEs	Adverse event(s)
ALK	Anaplastic lymphoma kinase
BBB	Blood–brain barrier
BM	Brain metastasis/brain metastases
BTB	Blood–tumor barrier
CI/CIs	Confidence interval(s)
CNS	Central nervous system
CNS-ORR	Central nervous system (intracranial) objective response rate
CNS-PFS	Central nervous system progression-free survival
DCE	Dynamic contrast-enhanced
DCE-MRI	Dynamic contrast-enhanced magnetic resonance imaging
DSC	Dynamic susceptibility contrast
DSC-MRI	Dynamic susceptibility contrast magnetic resonance imaging
DLT	Dose-limiting toxicity
DWI	Diffusion-weighted imaging
EGFR	Epidermal growth factor receptor
FLAIR	Fluid-attenuated inversion recovery
HIF-1α	Hypoxia-inducible factor 1 alpha
HSRT	Hypofractionated stereotactic radiotherapy
ICI/ICIs	Immune checkpoint inhibitor(s)
ILK	Integrin-linked kinase
ISRS	International Stereotactic Radiosurgery Society
K^trans^	Volume transfer constant
MBM	Melanoma brain metastases
MRI	Magnetic resonance imaging
NSCLC	Non-small-cell lung cancer
ORR	Objective response rate
OS	Overall survival
PAS	Periodic acid–Schiff
PD-1	Programmed cell death protein 1
PD-L1	Programmed death-ligand 1
PFS	Progression-free survival
PFS6	6-month progression-free survival
PET	Positron emission tomography
PS	Permeability–surface area product
rCBV	Relative cerebral blood volume
RN	Radiation necrosis
RT	Radiotherapy
SNO	Society for Neuro-Oncology
SRS	Stereotactic radiosurgery
SWI	Susceptibility-weighted imaging
TAZ	Transcriptional co-activator with PDZ-binding motif
TKI	Tyrosine kinase inhibitor
TNBC	Triple-negative breast cancer
VCO	Vessel co-option
VEGF	Vascular endothelial growth factor
VEGFR	Vascular endothelial growth factor receptor
VM	Vasculogenic mimicry
WBRT	Whole-brain radiotherapy
YAP	Yes-associated protein
